# STK33 alleviates gentamicin‐induced ototoxicity in cochlear hair cells and House Ear Institute‐Organ of Corti 1 cells

**DOI:** 10.1111/jcmm.13792

**Published:** 2018-09-06

**Authors:** Meijuan Zhou, Gaoying Sun, Lili Zhang, Guodong Zhang, Qianqian Yang, Haiyan Yin, Hongrui Li, Wenwen Liu, Xiaohui Bai, Jianfeng Li, Haibo Wang

**Affiliations:** ^1^ Otolaryngology‐Head and Neck Surgery Shandong Provincial Hospital Affiliated to Shandong University Jinan China; ^2^ Shandong Provincial Key Laboratory of Otology Jinan China; ^3^ Shandong Institute of Otolaryngology Jinan China

**Keywords:** apoptosis, extracellular signal‐regulated kinase 1/2, gentamicin, reactive oxygen species, serine/threonine kinase 33

## Abstract

Serine/threonine kinase 33 (STK33), a member of the calcium/calmodulin‐dependent kinase (CAMK), plays vital roles in a wide spectrum of cell processes. The present study was designed to investigate whether STK33 expressed in the mammalian cochlea and, if so, what effect STK33 exerted on aminoglycoside‐induced ototoxicity in House Ear Institute‐Organ of Corti 1 (HEI‐OC1) cells. Immunofluorescence staining and western blotting were performed to investigate STK33 expression in cochlear hair cells (HCs) and HEI‐OC1 cells with or without gentamicin treatment. CCK8, flow cytometry, immunofluorescence staining and western blotting were employed to detect the effects of STK33 knockdown, and/or U0126, and/or N‐acetyl‐L‐cysteine (NAC) on the sensitivity to gentamicin‐induced ototoxicity in HEI‐OC1 cells. We found that STK33 was expressed in both mice cochlear HCs and HEI‐OC1 cells, and the expression of STK33 was significantly decreased in cochlear HCs and HEI‐OC1 cells after gentamicin exposure. STK33 knockdown resulted in an increase in the cleaved caspase‐3 and Bax expressions as well as cell apoptosis after gentamicin damage in HEI‐OC1 cells. Mechanistic studies revealed that knockdown of STK33 led to activated mitochondrial apoptosis pathway as well as augmented reactive oxygen species (ROS) accumulation after gentamicin damage. Moreover, STK33 was involved in extracellular signal‐regulated kinase 1/2 pathway in primary culture of HCs and HEI‐OC1 cells in response to gentamicin insult. The findings from this work indicate that STK33 decreases the sensitivity to the apoptosis dependent on mitochondrial apoptotic pathway by regulating ROS generation after gentamicin treatment, which provides a new potential target for protection from the aminoglycoside‐induced ototoxicity.

## 1 INTRODUCTION

Hearing loss is regarded as a very common sensory disorder in humans all over the world, which seriously affects the quality of human life. In most cases, hearing loss is mainly attributable to hair cell (HC) damage caused by ototoxic drugs, of which aminoglycoside antibiotics, including kanamycin, amikacin, neomycin and gentamicin, are such a typical kind of drugs, with the potential to elicit toxic reactions to structures of the inner ear.^1^, ^2^ Although those drugs are widely used to treat gram‐negative bacterial infections, the ototoxic side effects have a great limitation in clinical use. Therefore, it is indispensable to better understand the underlying mechanism of aminoglycosides‐induced ototoxicity, which may, in turn, conduce to developing more effective therapies.

Serine/threonine kinase 33 (STK33) gene, which is identified in human chromosome 11 and mouse chromosome 7, encodes a serine/threonine kinase.^3^, ^4^ STK33 belongs to a member of the calcium/calmodulin‐dependent kinase (CAMK),^5^, ^6^ which is mainly existed in testis, certain brain regions and embryonic organs such as brain, heart and spinal cord in human and mouse and is likely to participate in spermatogenesis and organ ontogenesis.^7^, ^8^ Studies show that STK33 is co‐localized with the intermediate filament protein vimentin in tanycytes of mouse, rat and hamster,^9^ which is involved in the dynamic changes of the intermediate filament cytoskeleton assembly/disassembly, and regulates the cell structure as well as a few important functions through the specific phosphorylation of vimentin.^10^, ^11^ It is well known that apoptosis caused by STK33 suppression is mediated by the mitochondrial pathway, PI3K/AKT cascade or the mitogen‐activated protein kinases (MAPKs)‐signalling pathway.^6^

It has been documented that MAPKs, which are composed of a serine/threonine protein kinases, represent the classical signal transduction pathways.^12^, ^13^ Extracellular signal‐regulated kinase 1/2 (ERK1/2), one of the members of the MAPK family, is able to mediate many physiological and pathological processes, such as cell proliferation, protein synthesis, cell survival, differentiation, migration and apoptosis.^14^, ^15^, ^16^, ^17^ Previous studies have shown that activated ERK1/2 is present in both the cytoplasm and the nucleus,^18^ whereas inhibiting (MEK1/2) activation significantly reduces inner hair cells (IHCs) death caused by neomycin treatment, suggesting that ERK activation in supporting cells might promote HC death.^15^, ^18^ Moreover, ERK1/2 inhibition in cochlear explants causes a loss of outer hair cells (OHCs).^18^, ^19^ Of note, STK33‐retarded apoptosis is possibly linked to ERK1/2 pathway activation relevant to the accumulation of reactive oxygen species (ROSs).^13^

Available data have shown that the accumulation of ROSs is enhanced in HC after aminoglycoside exposure^20^ and the diverse antioxidants partially alleviated the aminoglycoside ototoxicity both in vitro and in vivo, suggesting a causal relationship between ROS production and HC death.^20^ Actually, ROS plays a crucial role in the promotion of apoptosis by interfering mitochondrial permeability, release of cytochrome c and caspases.^21^, ^22^ Thus, the accumulation of ROS is the primary molecular events in the process of HC death by activating multiple apoptotic pathways underpinning the ototoxicity of aminoglycosides.^1^, ^23^, ^24^, ^25^, ^26^

To date, the expression and function of STK33 in the mammalian inner ear have not been explored. Thus, the present study was designed to investigate whether STK33 expressed in cochlear HCs and House Ear Institute‐Organ of Corti 1 (HEI‐OC1) cells and, if so, what effect STK33 exerted on gentamicin‐induced damage in cochlear HCs and HEI‐OC1 cells, with special attention given to the possible relationship between STK33 and ERK1/2 signalling pathway.

## 2 MATERIALS AND METHODS

### 2.1 Animal experiments

All animal experiments were performed according to the protocols approved by the Animal Care Committee of Shandong University, PR China, and were in accordance with the Guide for the Care and Use of Laboratory Animal for Research Purposes. CBA mice were obtained by the reproduction in our laboratory. Post‐natal day (P)0 was defined as the day of birth. Mice models were set up by receiving a daily subcutaneous injection of gentamicin (200 mg/kg; G8170, Solarbio, Beijing, China) or sterile saline from P7 to P14. For each condition, at least, three male mice were included within each group and at least three individual experiments were conducted.

### 2.2 Cell culture

House Ear Institute‐Organ of Corti 1 cells were grown in high‐glucose DMEM (Gibco, Grand Island, NE, USA) supplemented with 10% foetal bovine serum (Gibco) at 33°C with 10% CO_2_. House Ear Institute‐Organ of Corti 1 cells were treated with 3 mmol/L gentamicin for 24 hour or were pre‐treated with 10 μmol/L MEK1/2 inhibitor (U0126, U120; Sigma, Saint Louis, USA) or 2 mmol/L ROS inhibitor (N‐acetyl‐L‐cysteine, NAC; A7250; Sigma, Saint Louis, USA) for 2 hour and then treated with 3 mmol/L gentamicin for 24 hour.

### 2.3 Cochlea dissection and culture

CBA mice at P4, P15, P30 and P60 were decapitated after anaesthesia, and the cochleae were carefully dissected out. The spiral ligament, stria vascularis and tectorial membrane were threw away, and then, basilar membranes were stuck to glass coverslips pre‐treated with Cell‐Tek (BD Biosciences, Franklin Lakes, NJ, USA). Finally, they were subjected to immunofluorescent staining analysis. The cochlear explants of P3‐P5 for primary culture were treated with or without 3 mmol/L gentamicin for 24 hour and, then, underwent immunofluorescent staining and western blotting analysis.

### 2.4 Auditory brainstem response (ABR) test

Auditory brainstem responses were measured at frequencies of 8, 12, 16, 24 and 32 kHz performed with Tucker‐Davis Technology (TDT) System hardware and software (Alachua, FL, USA) with 1024 stimulus repetitions per record. Mice were anesthetized with a chloral hydrate and prepared for ABR test. The record electrode was inserted into subcutaneous tissue at the vertex, and reference and ground electrodes were, respectively, placed subcutaneously at ipsilateral ear and thigh. Sound stimuli were presented to the left ear through a metal loudspeaker in the external auditory meatus. Auditory brainstem response waveforms were recorded in 10‐dB intervals down from the maximum amplitude until no waveform could be visualized.

### 2.5 Frozen section

Tissues from CBA mice were dissected after anaesthesia and fixed with 4% paraformaldehyde (PFA) in PBS at 4°C overnight. Then, they were decalcified and then dehydrated by successive 15%, 20% and 30% sucrose in 1× PBS. They were embedded in OCT compound (Tissue‐Tek, Sakura Finetek, Torrance, USA) and sliced into the thickness of 7‐μm sections performed with a cryostat (Leica CM 1850, Nussloch, Germany).

### 2.6 Immunofluorescence staining

The samples were fixed with 4% PFA in PBS for 1 hour, permeabilized with 1% Triton X‐100 in PBS for 30 minutes and then blocked with 5% bovine serum albumin, 0.1% Triton X‐100 and 0.02% sodium azide in PBS (PBT‐1) for 1 hour. Then, they were incubated in PBT‐1 overnight at 4°C with the following primary antibodies: anti‐STK33 (1:50 dilution, SAB1409680; Sigma, Saint Louis, USA), anti‐p‐ERK1/2 (1:500 dilution, 4370s; CST, Danvers, USA), anti‐cleaved caspase‐3 (1:500 dilution, 9664s; CST, Danvers, USA), anti‐myosin 7a (1:1000 dilution, 25‐6790; Proteus Biosciences, Ramona CA, USA; 1:500 dilution, 138‐1‐c; DSHB,Iowa, IA, USA). The next day, the samples were incubated with the secondary fluorescent antibodies (1:1000 dilution; Invitrogen, Carlsbad, Iowa, CA, USA) along with DAPI (1:1000 dilution, D9542; Sigma, Saint Louis, USA) in 1% bovine serum albumin and 0.1% Triton X‐100 in PBS for 1 hour in dark at room temperature. The samples were observed under a laser scanning confocal microscope (Leica).

DCFH‐DA (D6883; Sigma, Saint Louis, USA) staining was used to detect ROS generation. Samples were incubated in DMEM culture medium containing DCFH‐DA for 30 minutes at 37°C in dark. After staining, the samples were washed in PBS and imaged with the fluorescent microscope (Leica). The Click‐iT^®^ Plus TUNEL Assay (Life Technologies, Oregon, USA) was used to detect cell apoptosis according to the protocol.

### 2.7 Protein extraction and western blotting

House Ear Institute‐Organ of Corti 1 cells and tissues were lysed with cold RIPA lysis buffer (P0013B; Beyotime Institute of Biotechnology, Shanghai, China) plus protease inhibitor cocktail (P8340; Sigma) for 30 minutes at 4°C and then centrifuged at 13201 g for 20 minutes at 4°C. Protein concentrations were detected by the BCA Protein Assay Kit (Shenergy Biocolor Bioscience & Technology Company, Shanghai, China). The same amount of protein sample was denatured and separated by 10% SDS‐PAGE. Then, the proteins were transferred to polyvinylidene difluoride membranes (Immobilon‐P, IPVH00010; Millipore, Darmstadt, Germany). The membranes were blocked in Tris‐buffered saline and 0.05% Tween 20 (TBST) containing 5% non‐fat dried milk for 2 hour at room temperature and then were incubated with the primary antibodies in TBST containing 3% non‐fat dried milk at 4°C overnight. The primary antibodies consist of anti‐STK33 (1:500 dilution, SAB1409680; Sigma, Saint Louis, USA), anti‐p‐ERK1/2 (1:500 dilution, 4370s; CST, Danvers, USA), anti‐cleaved caspase‐3 (1:500 dilution, 9664s; CST, Danvers, USA), anti‐Bax (1:500 dilution, ab32503; Abcam, Cambridge, MA, USA), anti‐β‐actin (1:1000 dilution; ZSGB‐BIO, Beijing, China) and anti‐GAPDH (1:1000 dilution; ZSGB‐BIO, Beijing, China). The next day, the membranes were incubated with the secondary antibodies for 1 hour at room temperature. Finally, the protein signals were detected using an ECL kit (Millipore, Billerica, MA, USA) and analysed using ImageJ software.

### 2.8 siRNA transfection in HEI‐OC1 cells

STK33‐specific mouse siRNA (GenePharma, Shanghai, China) was obtained to knock down the expression of STK33 in HEI‐OC1 cells. The siRNA encoding a nonsense sequence was used for the negative control. House Ear Institute‐Organ of Corti 1 cells were seeded in six‐well plates to make sure 50% confluence after 24 hour. Transfection was finished according to the manufacturer's instructions. After 6 hour, the Opti‐MEM (Gibco BRL) medium was replaced with DMEM containing foetal bovine serum. The negative control siRNA or siRNA‐STK33 was mixed with 10 μL Lipofectamine 3000 (Invitrogen) at a final concentration of 500 nmol/L siRNA in medium. Finally, cells were collected for western blotting, immunofluorescence staining, CCK8 assay and flow cytometry. All siRNA sequences were as follows:

siRNA‐negative control sense 5′‐UUCUCCGAACGUGUCACGUTT‐3′, antisense 5′‐ACGUGACACGUUCGGAGAATT‐3′; siRNA‐STK33‐1490 sense 5′‐CCAGUCUGGGAAUCUGUAATT‐3′, antisense 5′‐UUACAGAUUCCCAGACUGGTT‐3′.

### 2.9 Cell viability

Cell Counting Kit‐8 (CCK8) was used to assess the cell viability. House Ear Institute‐Organ of Corti 1 cells were cultured at the density of 3000 cells/well in a 96‐well plate. Cells were transfected with siRNA‐STK33 or siRNA‐control for 48 hour. CCK8 (96992; Sigma, Saint Louis, USA) was added to per well and reacted for 2 hour. The optical density (OD) values were measured at 450 nm using an ELISA reader (Multiscan MK3).

### 2.10 Flow cytometry

A FITC Annexin V Apoptosis Detection Kit I (556547; BD, RUO) was used to detect apoptosis by flow cytometry. House Ear Institute‐Organ of Corti 1 cells were trypsinized, collected and washed twice with cold PBS and then resuspended gently in 1× binding buffer at a concentration of 1 × 10^6^ cells/mL. At room temperature, 5 μL Annexin V‐FITC and 5 μL propidium iodide (PI) were added to cell suspension and incubated in the dark for 15 minutes. The cells were immediately detected by flow cytometry and then analysed with the FlowJo 7.6 software.

### 2.11 Statistical analyses

Data were presented as mean ± SD. *T* test was applied for comparisons between two groups, and one‐way ANOVA was used to compare more than two groups. *P* < 0.05 was considered statistically significant.

## 3 RESULTS

### 3.1 STK33 is expressed in the cochlea and HEI‐OC1 cells

**Figure 1:**
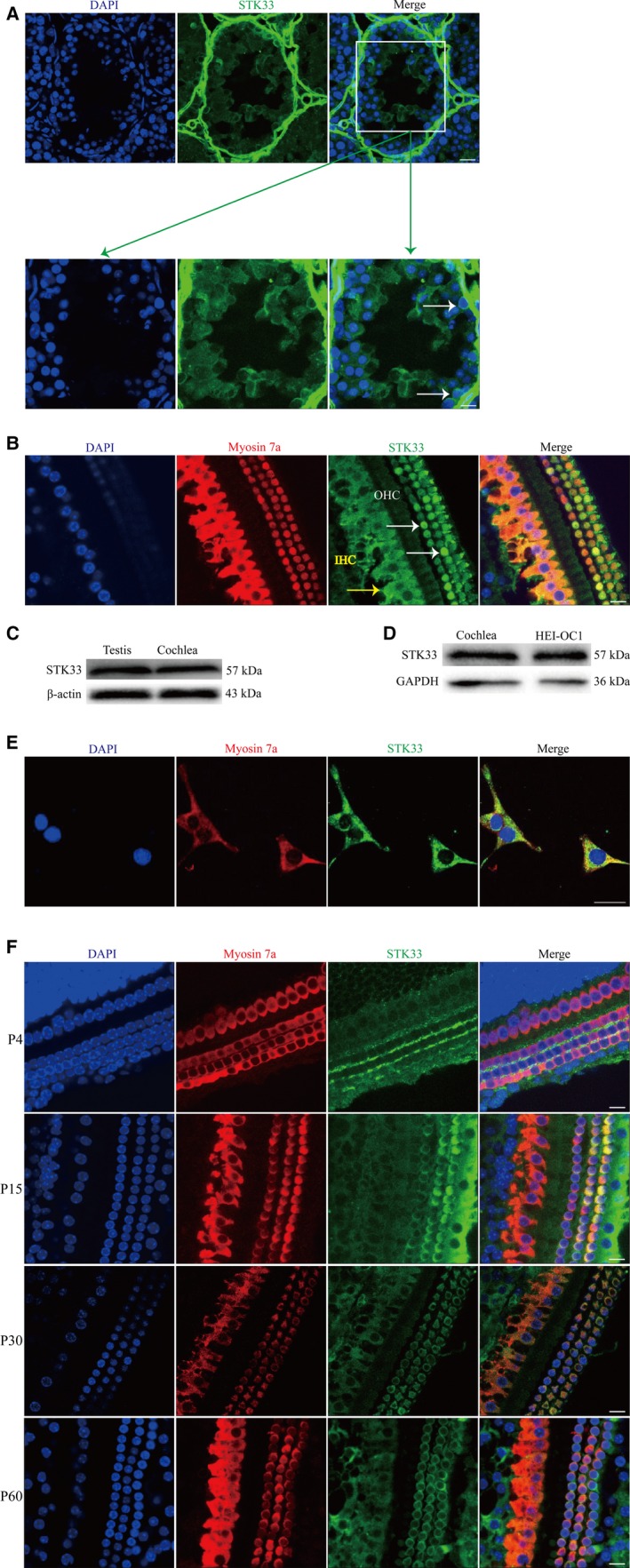
STK33 Expression in the Cochlear Hair Cells (HCs) and HEI‐OC1 Cells. A, Positive control. Immunofluorescence staining showed STK33 expression in the cells of testis (white arrow). B, Representative images of STK33 (green) expression in P30 cochlear HCs by immunofluorescence staining (IHCs, yellow arrow, and OHCs, white arrow). Myosin 7a (red) was used as HC marker. C, Western blotting results showed that STK33 expression in CBA cochlea was consistent with that in testis. D, Western blotting results showed that STK33 was expressed in HEI‐OC1 cells. E, Immunofluorescence staining showed STK33 expression in HEI‐OC1 cells. F, Immunofluorescence staining showed the expression pattern of STK33 in the middle turn of mouse cochlea. At P4, STK33 (green) was expressed in IHCs and the intercellular space of OHCs. At P15, STK33 (green) expression was found in OHCs and IHCs. From P30, STK33 (green) was highly expressed in OHCs and IHCs. Myosin 7a (red) was used as HEI‐OC1 cells marker. Scale bars = 30 μm. IHCs, inner hair cells; OHCs, outer hair cells; HEI‐OC1, House Ear Institute‐Organ of Corti 1; STK33, serine/threonine kinase 33

Hair cells were marked by myosin 7a which was usually used as HCs markers.^27^ As shown in Figure 1B,C, STK33 was strongly expressed in OHCs and IHCs in the P30 cochlea by immunofluorescent staining and western blotting, which was consistent with the expression in testis served as the positive control (Figure 1A). And STK33 expression was found in HEI‐OC1 cells by western blotting and immunofluorescence staining (Figure 1D,E).

Immunofluorescence staining showed the expression pattern of STK33 in the middle turn of mouse cochlea. At P4, STK33 was expressed in IHCs and the intercellular space of OHCs (Figure 1F). At P15, STK33 expression was found in OHCs and IHCs (Figure 1F). From P30, STK33 was highly expressed in OHCs and IHCs (Figure 1F). These results suggested that STK33 was expressed in a specific manner in post‐natal mouse cochlea.

### 3.2 STK33 expression in cochlear HCs is decreased after gentamicin treatment and mitochondrial apoptosis is activated

**Figure 2:**
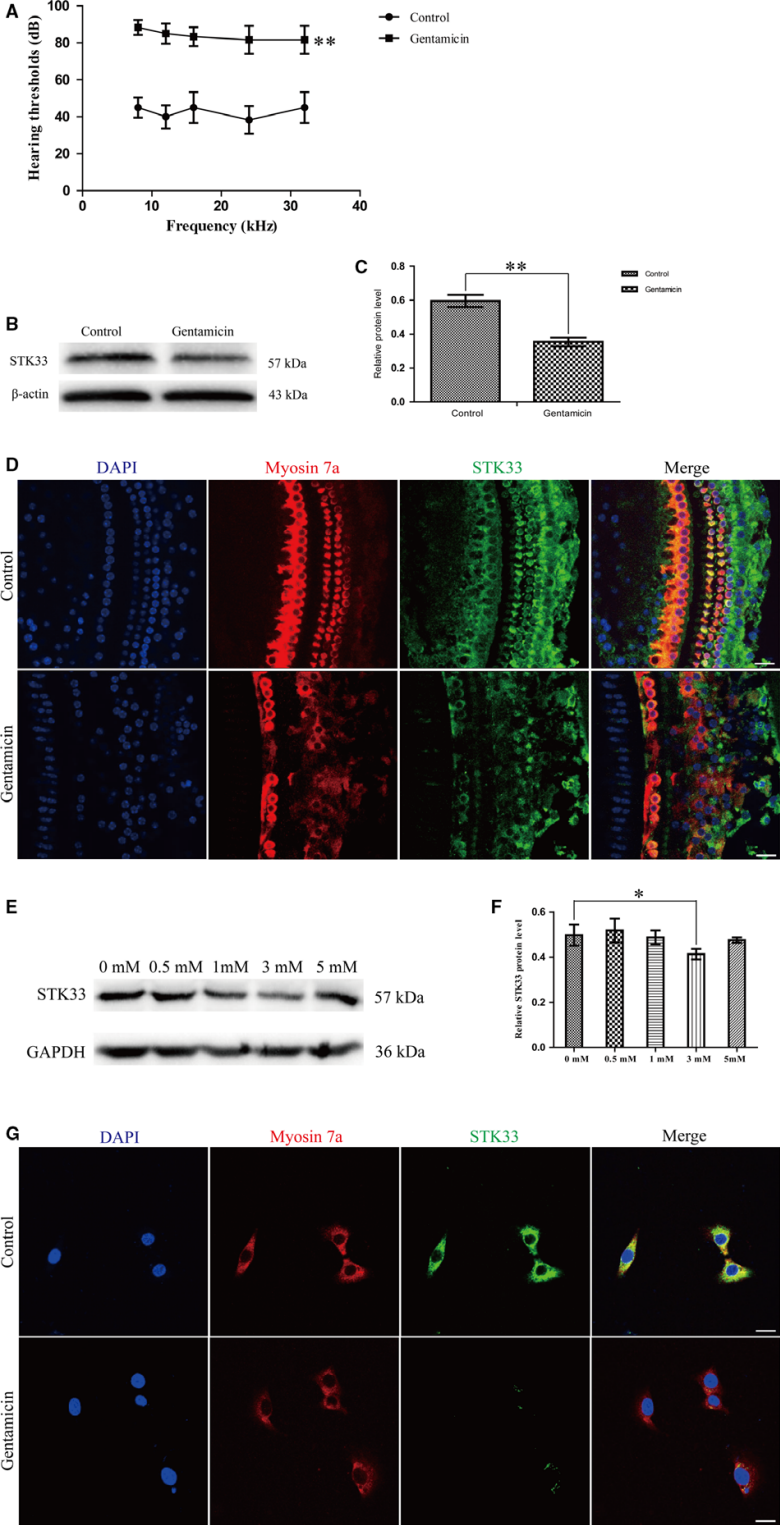
STK33 Expression was Down‐Regulated in Cochlear Hair Cells (HCs) and HEI‐OC1 Cells After Gentamicin Treatment. CBA mice were subcutaneously injected gentamicin (200 mg/kg) from P7 to P14. The control group was injected sterile saline. A, ABR thresholds were increased after gentamicin treatment compared to the control ones at 5‐6 wk. **P < 0.01, n = 3. B‐C, Western blotting results confirmed that the expression level of STK33 was decreased in cochlear HCs after gentamicin treatment compared to the control group at 5‐6 wk. **P < 0.01, n = 3. D, STK33 expression in cochlear HCs was decreased after gentamicin treatment compared to the control ones by immunofluorescence staining at 5‐6 wk. Scale bars = 30 μm. E‐F, Western blotting results demonstrated that the expression of STK33 protein was significantly reduced in HEI‐OC1 cells with 3 mmol/L gentamicin treatment for 24 h compared to the untreated controls. GAPDH was served as the control. *P < 0.05, n = 3. G, STK33 (green) expression was decreased in HEI‐OC1 cells after gentamicin treatment compared to the untreated controls by immunofluorescence staining. Myosin 7a (red) was used as the marker in HEI‐OC1 cells. Scale bar = 30 μm. ABR, auditory brainstem response; HEI‐OC1, House Ear Institute‐Organ of Corti 1; STK33, serine/threonine kinase 33

To explore whether STK33 expression makes a difference in cochlear HCs after gentamicin exposure, CBA mice were chose to subcutaneously inject gentamicin (200 mg/kg) from P7 to P14. The hearing of mice at 5‐6 weeks was examined by ABR test. The results showed that the ABR threshold shifts of gentamicin‐treated mice were increased compared to that of the control ones (Figure 2A), which suggested that gentamicin can cause hearing loss. Western blotting results confirmed that STK33 was decreased after gentamicin treatment, compared to the control group (Figure 2B,C). Immunofluorescence staining results showed that the expression of STK33 was reduced in cochlear HCs with gentamicin exposure compared to the control (Figure 2D). And we found that STK33 was significantly reduced in HEI‐OC1 cells with 3 mmol/L gentamicin treatment for 24 hour by western blotting compared to the untreated control (Figure 2E,F). Immunofluorescence staining results also showed that STK33 expression was decreased in HEI‐OC1 cells exposed to 3 mmol/L gentamicin for 24 hour compared to the untreated control (Figure 2G). These results suggested that STK33 expression was decreased in cochlear HCs and HEI‐OC1 cells after gentamicin damage.

**Figure 3:**
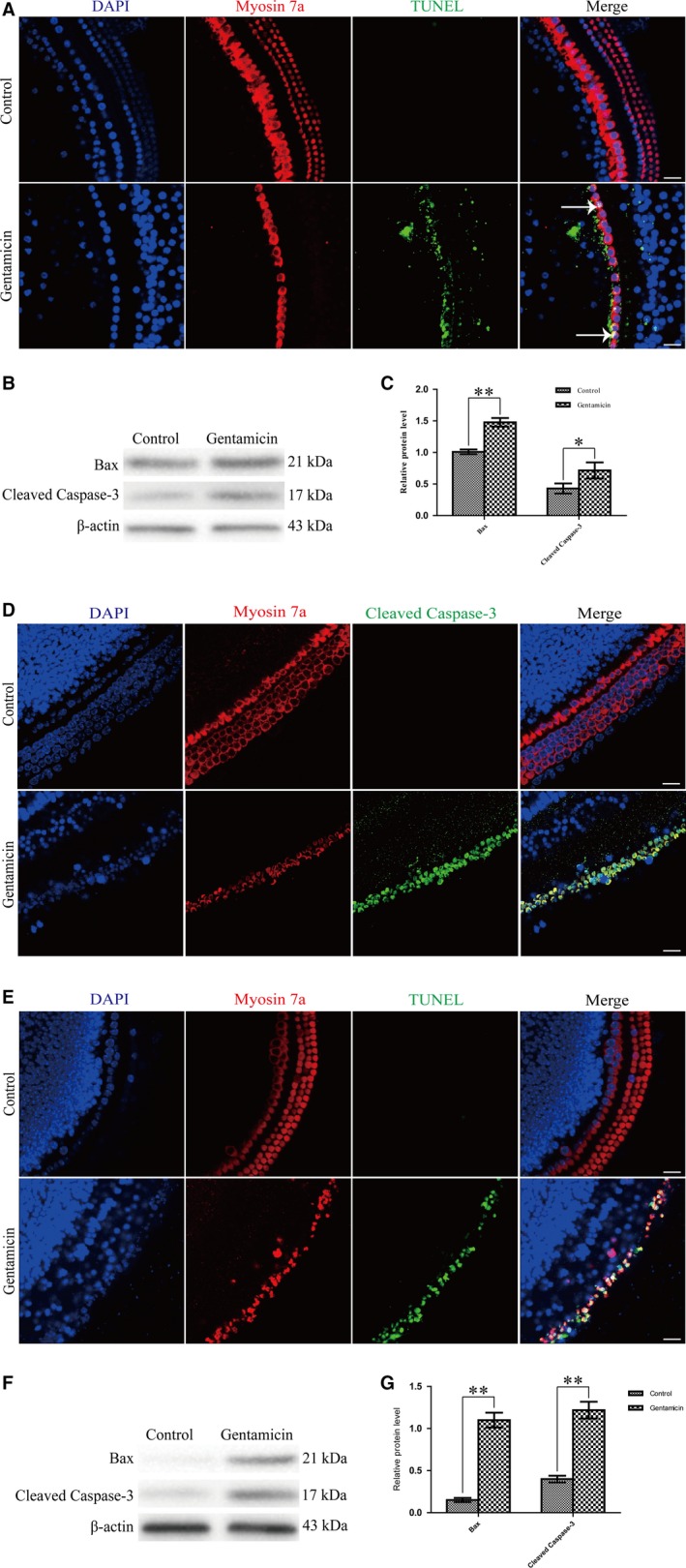
Mitochondrial Apoptosis was Activated in Cochlear HCs After Gentamicin Treatment. A, Immunofluorescence staining results showed that TUNEL‐positive nuclei (white arrow) were increased in gentamicin‐induced hearing loss group compared to the control group in vivo experiment. Scale bars = 30 μm. B‐C, Western blotting results confirmed that the expression levels of Bax and cleaved caspase‐3 were increased in gentamicin‐induced hearing loss group compared to the control group. *P < 0.05, **P < 0.01, n = 3. D‐E, Immunofluorescence staining results showed that in vitro experiment, the expressions of cleaved caspase‐3 and TUNEL‐positive nuclei were increased after gentamicin damage compared to the untreated controls in primary culture of cochlear HCs. Scale bars = 30 μm. F‐G, Western blotting results confirmed that in primary culture of cochlear HCs, the expression levels of Bax and cleaved caspase‐3 protein were increased after gentamicin treatment compared to the untreated controls. **P < 0.01, n = 3. HC, hair cell; HEI‐OC1, House Ear Institute‐Organ of Corti 1; STK33, serine/threonine kinase 33

We found that the positive stainings of TUNEL were increased in IHCs of gentamicin‐treated mice compared to the control ones in vivo experiment (Figure 3A). Western blotting results confirmed that the protein expression levels of Bax and cleaved caspase‐3 (an activated form of caspases‐3) were significantly increased compared to the controls (Figure 3B,C). In vitro experiment, the expressions of cleaved caspase‐3 and TUNEL‐positive nuclei were increased after gentamicin damage by immunofluorescence staining compared to the untreated controls in the primary culture of cochlear HCs (Figure 3D,E), and the protein expressions of Bax and cleaved caspase‐3 were significantly increased after gentamicin damage by western blotting (Figure 3F,G). Together, these results implied that STK33 down‐regulation might activate the mitochondrial apoptosis in cochlear HCs after gentamicin damage.

### 3.3 Knockdown of STK33 increases the cell apoptosis in HEI‐OC1 cells after gentamicin damage

**Figure 4:**
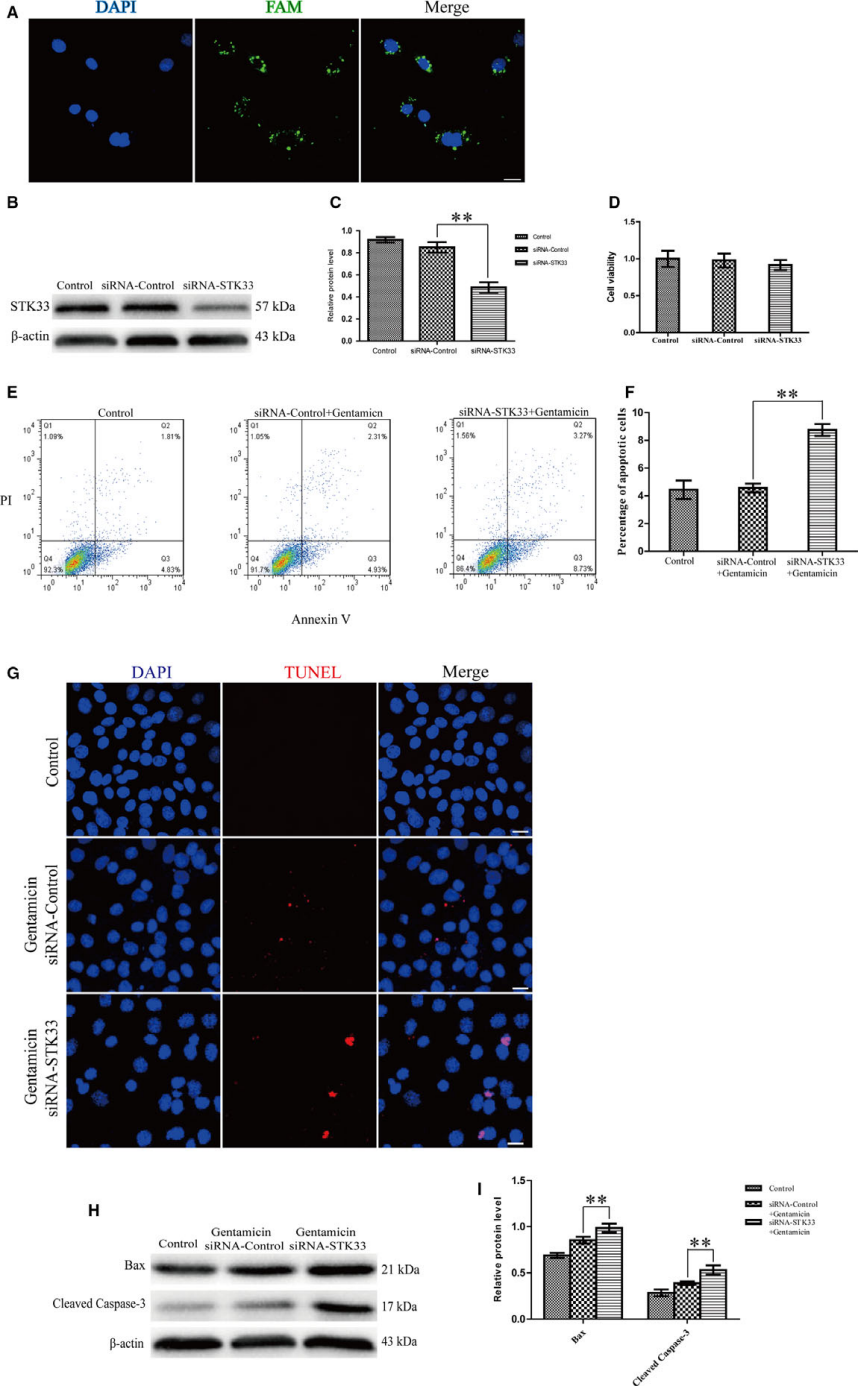
Knockdown of STK33 Increased the Apoptosis in HEI‐OC1 Cells After Gentamicin Treatment. A, Representative images of the transfection efficiency were measured by using nonsense siRNA conjugated with 6′‐carboxyfluorescein (FAM). Scale bars = 30 μm. B‐C, Western blotting results confirmed that transfection of siRNA‐STK33 was successful. **P < 0.01, n = 3. D, Knockdown of STK33 had little decrease in the cell viability in HEI‐OC1 cells of siRNA‐STK33 transfection compared to the siRNA‐Control ones by CCK8 assay. E‐F, After gentamicin exposure, the apoptotic cells were increased in HEI‐OC1 cells transfected with siRNA‐STK33 compared to siRNA‐Control ones by flow cytometry. G, Immunofluorescence staining result demonstrated that the TUNEL‐positive cells were increased in the siRNA‐STK33 transfection group compared to the siRNA‐Control transfection group after gentamicin damage. Scale bars = 30 μm. H‐I, Western blotting results showed that the protein expression levels of Bax and cleaved caspase‐3 were increased in the siRNA‐STK33 transfection group compared to the siRNA‐Control transfection group after gentamicin treatment. **P < 0.01, n = 3. HEI‐OC1, House Ear Institute‐Organ of Corti 1; STK33, serine/threonine kinase 33

To further explore the role of STK33 in HEI‐OC1 cells apoptosis for gentamicin‐induced damage, STK33 was knocked down by siRNA transfection. House Ear Institute‐Organ of Corti 1 cells without any treatment were regarded as the control group, and HEI‐OC1 cells transfected with nonsense siRNA were regarded as the siRNA‐Control group. The efficiency of the transfection system was measured using nonsense siRNA conjugated with 6′‐carboxyfluorescein (FAM), and the results showed a successful transfection by immunofluorescence staining (Figure 4A). Western blotting results showed that the protein expression of STK33 was successfully silenced in siRNA‐STK33‐treated cells, but had no change in the siRNA‐Control ones (Figure 4B,C). These results showed that transfection of siRNA‐STK33 effectively inhibited STK33 expression in HEI‐OC1 cells compared to the siRNA‐Control ones.

Additionally, down‐regulation of STK33 caused little reduction in the HEI‐OC1 cell viability by CCK8 assay (Figure 4D). After gentamicin exposure, however, the apoptosis was significantly enhanced in siRNA‐STK33 transfection group by flow cytometry compared to the siRNA‐Control group (Figure 4E,F). These results suggested that HEI‐OC1 cells of STK33 down‐regulation were more sensitive to gentamicin‐induced apoptosis. Furthermore, after gentamicin exposure, the positive stainings of TUNEL were enhanced in the siRNA‐STK33 transfection group compared to the siRNA‐Control transfection group (Figure 4G). Western blotting showed that the protein expression levels of Bax and cleaved caspase‐3 were increased in the siRNA‐STK33 transfection group compared with the siRNA‐Control group after gentamicin treatment (Figure 4H,I). These results demonstrated that the down‐regulation of STK33 can increase apoptosis in HEI‐OC1 cells co‐treated with gentamicin.

### 3.4 STK33 expression is reduced by U0126 treatment which inhibits p‐ERK1/2 expression in cochlear HCs and HEI‐OC1 cells after gentamicin stimulus

**Figure 5:**
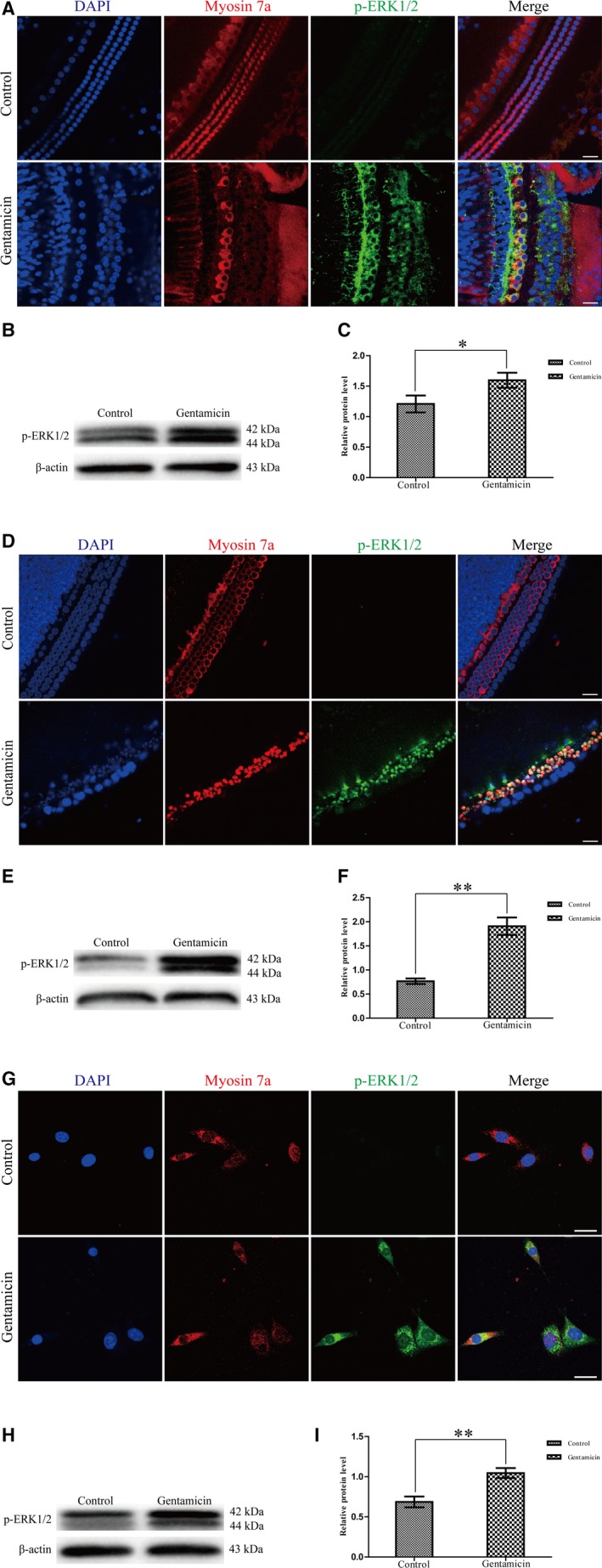
p‐ERK1/2 Expression was Increased in Cochlear HCs and HEI‐OC1 Cells After Gentamicin Damage. Expression of p‐ERK1/2 was increased with gentamicin exposure compared to the untreated control by immunofluorescence staining and western blotting in cochlear HCs (A‐C), the primary culture of cochlear HCs (D‐F) and HEI‐OC1 cells (G‐I). *P < 0.05, **P < 0.01, n = 3. Scale bars = 30 μm. ERK1/2, extracellular signal‐regulated kinase 1/2; HC, hair cell; HEI‐OC1, House Ear Institute‐Organ of Corti 1

We found that the expression of p‐ERK1/2 was significantly increased in cochlear HCs and HEI‐OC1 cells compared to the untreated controls by immunofluorescence staining and western blotting (Figure 5A‐I). This suggested ERK pathway was activated in gentamicin‐induced HC apoptosis.

**Figure 6:**
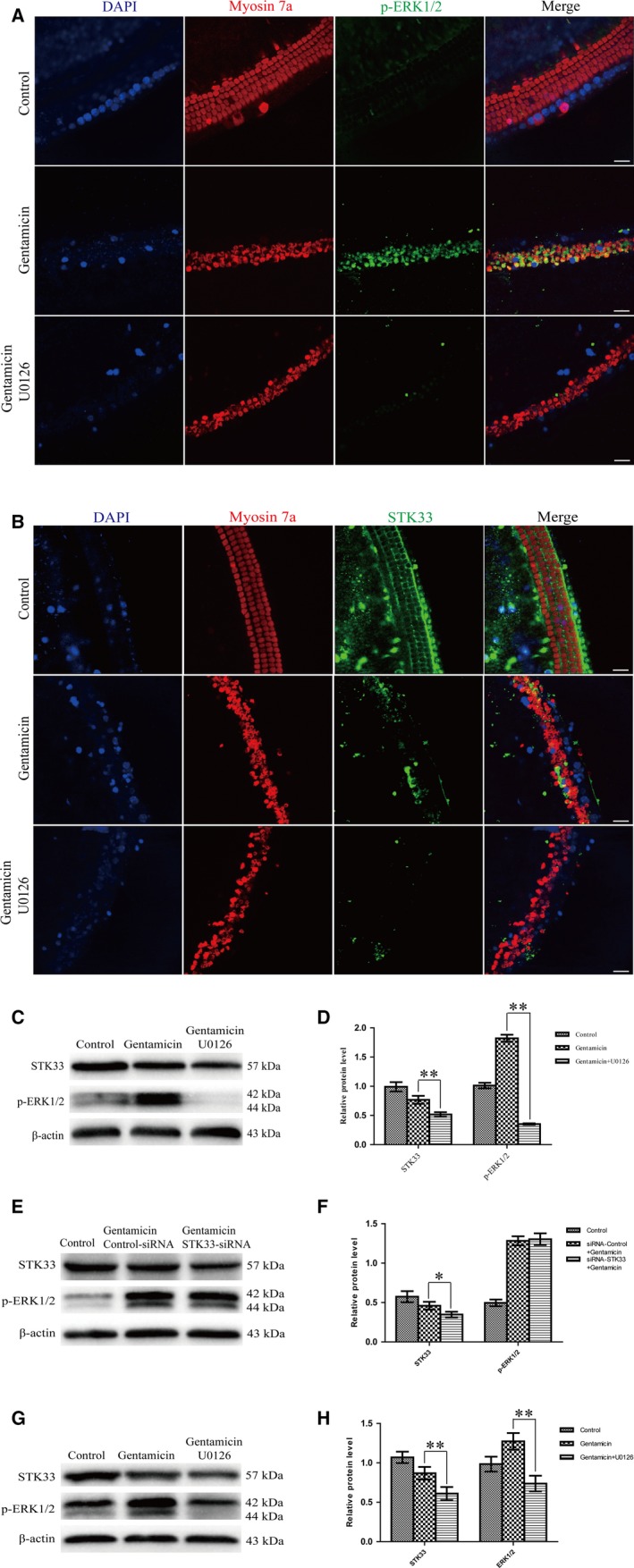
STK33 Expression was Decreased by U0126 Treatment in Primary Culture of Cochlear HCs and HEI‐OC1 Cells After Gentamicin Damage. Hair cells (HCs) were pre‐incubated with 10 μmol/L U0126 for 2 h, followed by treating with 3 mmol/L gentamicin for 24 h. The expression of STK33 was significantly reduced by U0126 which inhibited p‐ERK1/2 expression after gentamicin treatment in the primary culture of cochlear HCs by immunofluorescence staining (A‐B) and western blotting (C‐D). **P < 0.01, n = 3. Scale bars = 30 μm. E‐F, Western blotting results showed that knockdown STK33 by transfected with siRNA‐STK33 had no effect on p‐ERK1/2 expression compared to the siRNA‐Control transfection one after gentamicin treatment in HEI‐OC1 cells. G‐H, The expression level of STK33 was significantly decreased in HEI‐OC1 cells by U0126 which inhibited p‐ERK1/2 expression after gentamicin treatment by western blotting. **P < 0.01, n = 3. ERK1/2, extracellular signal‐regulated kinase 1/2; HEI‐OC1, House Ear Institute‐Organ of Corti 1; STK33, serine/threonine kinase 33

To further explore the relationship of STK33 and ERK1/2 signalling pathway, the expression of STK33 and p‐ERK1/2 in cochlear HCs and HEI‐OC1 cells in response to U0126 which is a specific inhibitor of the ERK1/2 signalling pathway was examined by immunofluorescence staining and western blotting. Immunofluorescence staining results showed that U0126 inhibited p‐ERK1/2 expression as well as STK33 expression after gentamicin treatment in primary culture of cochlear HCs compared to the untreated controls (Figure 6A,B). Meanwhile, the protein expression levels of STK33 and p‐ERK1/2 were significantly reduced by western blotting after exposed to U0126 with gentamicin compared to the ones treated with gentamicin only in primary culture of cochlear HCs (Figure 6C,D). These data implied that STK33 might be involved in the ERK1/2 signalling pathway. Moreover, we found that the protein expression of p‐ERK1/2 remained unchanged in the siRNA‐STK33 transfection of HEI‐OC1 cells compared with the siRNA‐Control transfection ones after gentamicin damage by western blotting (Figure 6E,F). But STK33 expression was significantly decreased in HEI‐OC1 cells by U0126 treatment after gentamicin damage compared with only gentamicin treatment by western blotting (Figure 6G,H). Together, down‐regulation of STK33 only markedly suppressed STK33 expression but failed to impact p‐ERK1/2 expression; however, U0126 significantly reduced both p‐ERK1/2 and STK33 expressions in HEI‐OC1 cells. These results indicated that STK33 might be regulated by ERK1/2.

### 3.5 NAC treatment successfully rescues cell apoptosis induced by STK33 down‐regulation in cochlear HCs and HEI‐OC1 cells after gentamicin stimulus

**Figure 7:**
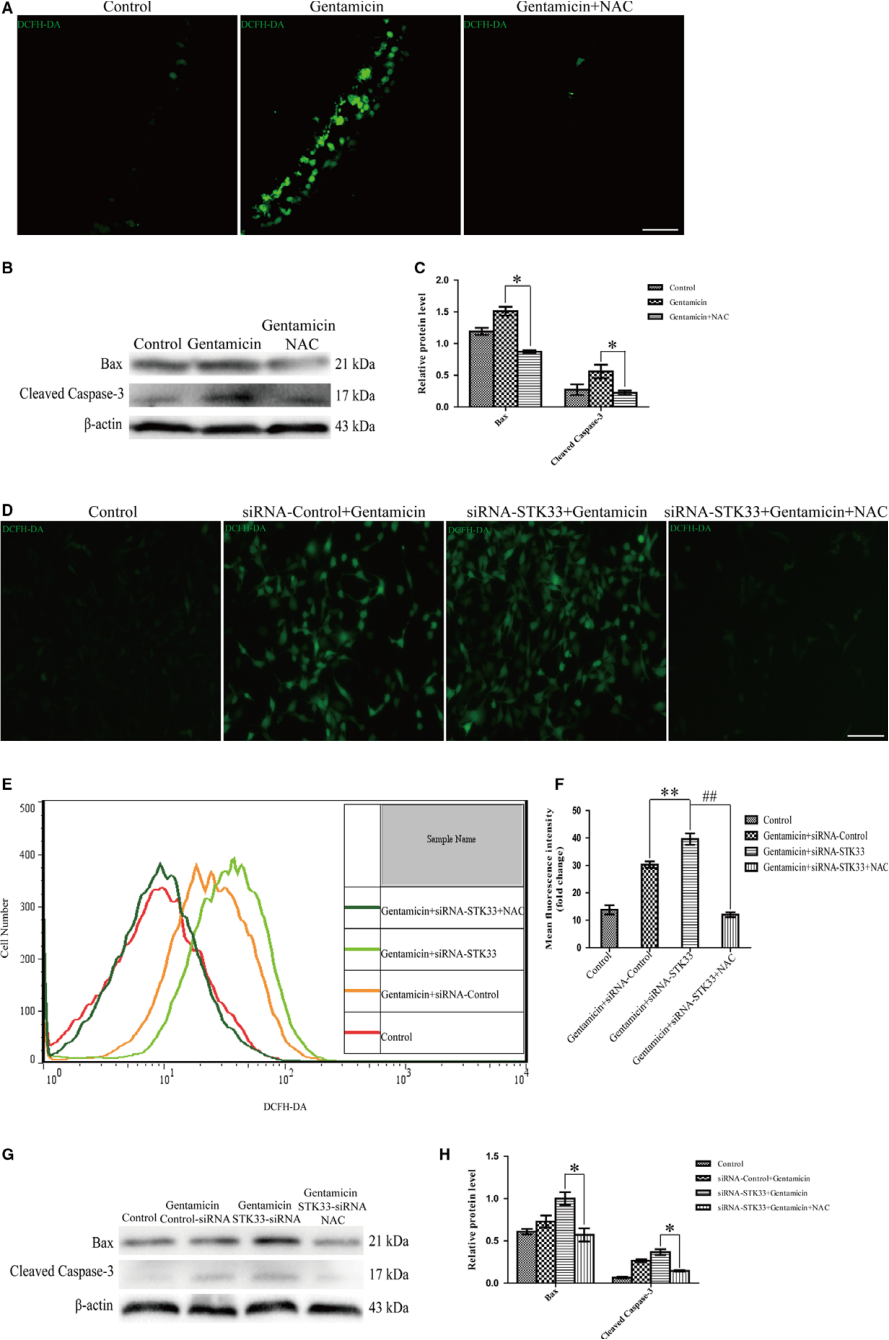
NAC Treatment Successfully Rescues Cell Apoptosis Induced by STK33 Down‐Regulation in Cochlear HCs and HEI‐OC1 Cells After Gentamicin Co‐Stimulus. Hair cells (HCs) and transfection of siRNA‐STK33 HEI‐OC1 cells were pre‐incubated with 2 mmol/L NAC for 2 h, followed by treating with 3 mmol/L gentamicin for 24 h. A, The intracellular ROS level was increased after gentamicin treatment by DCFH‐DA staining compared to the untreated control. But the intracellular ROS level was decreased by NAC treatment after gentamicin damage in cochlear HCs by DCFH‐DA staining. Scale bars = 30 μm. B‐C, Western blotting results demonstrated that the expression levels of Bax and cleaved caspase‐3 were decreased by NAC co‐treatment with gentamicin compared to the gentamicin treatment in cochlear HCs. *P < 0.05, n = 3. D‐F, The ROS levels were increased in HEI‐OC1 cells transfected of siRNA‐STK33 compared to transfected of siRNA‐control ones after gentamicin treatment by DCFH‐DA staining and flow cytometry, but NAC had the opposite role in the ROS levels. **P < 0.01, ^#*#*^ *P* < 0.01, n = 3. Scale bars = 50 μm. G‐H, Western blotting results demonstrated that the expression levels of Bax and cleaved caspase‐3 were significantly decreased in HEI‐OC1 cells transfection of siRNA‐STK33 in combination with NAC pre‐treatment after gentamicin treatment compared to transfection of siRNA‐STK33. *P < 0.05, n = 3. HEI‐OC1, House Ear Institute‐Organ of Corti 1; NAC, N‐acetyl‐L‐cysteine; ROS, reactive oxygen species; STK33, serine/threonine kinase 33

It is reported that ROS is involved in aminoglycoside‐induced HC damage.^2^, ^23^, ^28^ The intracellular ROS level was increased after gentamicin treatment by DCFH‐DA staining compared with the untreated control in primary culture of cochlear HCs, but NAC inhibited the intracellular ROS level after gentamicin damage (Figure 7A). As shown in Figure 7B,C, the expression levels of Bax and cleaved caspase‐3 were significantly reduced in pre‐treatment of NAC co‐treatment with gentamicin group compared with the gentamicin treatment group by western blotting. These results suggested that the mitochondrial apoptosis was inhibited by NAC treatment after gentamicin damage in cochlear HCs.

To determine whether the up‐regulation of ROS levels conduced to the increase in sensitivity exposed to gentamicin after STK33 knockdown in HEI‐OC1 cells, NAC was used to treat the HEI‐OC1 cells. We found that the ROS levels were significantly increased in HEI‐OC1 cells transfection of siRNA‐STK33 after gentamicin treatment compared with the siRNA‐Control ones by immunofluorescence staining and flow cytometry (Figure 7D‐F). These results indicated that knockdown of STK33 significantly increased intracellular ROS levels after gentamicin stimulus. Furthermore, we found that the expression levels of Bax and cleaved caspase‐3 protein were significantly decreased by NAC treatment with HEI‐OC1 cells transfection of siRNA‐STK33 compared with transfection of siRNA‐STK33 ones by western blotting (Figure 7G,H). These results suggested that STK33 knockdown increased the sensitivity to mitochondrial apoptosis by activating ROS generation after gentamicin treatment.

## 4 DISCUSSION

Previous studies have confirmed that STK33 is highly expressed in testis,^5^ and in this work, positive immunostaining was found in testis, mouse cochlea and HEI‐OC1 cells, whereby indicating that STK33 truly exists in the cochlea and HEI‐OC1 cells. Moreover, in the post‐natal cochlea, STK33 is specifically expressed in HCs and the expression pattern was different in different time points. Thus, we speculate that STK33 might play a role in the maturation and differentiation of the post‐natal mouse cochlea.

While gentamicin is widely used in clinics for treating bacterial infections, the side effect of ototoxicity greatly limits their clinical use. In the present study, mice treated with gentamicin showed elevated ABR thresholds in all frequencies examined and severe HC loss, indicating that an animal model with hearing impairment has been set up successfully, which is conductive to the subsequent experiments. Interestingly, we found that in both cochlear HCs and HEI‐OC1 cells the expression of STK33 was markedly decreased after gentamicin exposure, suggesting that STK33 exerts an otoprotective role in normal cochlear HCs and HEI‐OC1 cells.

It has been well established that HC apoptosis is the predominant mechanism underlying the aminoglycoside‐induced HC damage and mitochondrial apoptotic pathway is involved in the HC apoptosis. In our work, TUNEL‐positive cells and the expressions of Bax, cleaved caspase‐3 were significantly increased in cochlear HCs in vivo and in vitro after gentamicin treatment. This indicates that gentamicin exerting its cytotoxicity on HCs is mainly attributable to the induction of apoptosis via mitochondrial pathway.

As STK33 suppression in other tissues can cause cell apoptosis and this effect is mainly mediated by mitochondrial pathway, we subsequently explored the effect of STK33 on gentamicin sensitivity in HEI‐OC1 cells. siRNA‐STK33 was generated and introduced into HEI‐OC1 cells, and we found that only HEI‐OC1 cells transfected with siRNA‐STK33 exhibited a marked reduction in protein levels of STK33, suggesting that STK33 is successfully down‐regulated in HEI‐OC1 cells. We also observed that after gentamicin exposure, knockdown of STK33 remarkably increased HEI‐OC1 cells apoptosis as evidenced by flow cytometry and TUNEL‐positive staining. These results suggest that, after gentamicin stimulus, STK33 down‐regulation might render HEI‐OC1 cells more sensitive to apoptosis. It is well known that caspases are the key proteins that modulate the apoptotic response and the activated caspases, especially, the caspase‐3, which can cleave many cellular substrates, eventually leading to apoptosis. In the present study, we found that cleaved caspase‐3 was significantly increased in STK33 knockdown cells after gentamicin treatment, suggesting that the caspase‐dependent pathway is activated in this process. Evidence has confirmed that the Bcl‐2 family, which generally either represses apoptosis or promotes apoptosis, plays a key role in controlling the activation of caspases.^29^, ^30^ In this work, we demonstrated that Bax was dramatically increased in STK33 knockdown cells after gentamicin exposure, indicating that STK33 knockdown increases the gentamicin‐induced apoptosis in HEI‐OC1 cells, at least involved in up‐regulating proapoptotic Bax.

Previous studies have demonstrated that MAPKs are important mediators in gentamicin‐induced HC apoptosis,^31^, ^32^ and ERKs are a vital member of the MAPK family.^14^, ^15^ We found that in cochlear HCs, cochlear explants and HEI‐OC1 cells, the expression of p‐ERK1/2 was markedly increased after gentamicin insult, suggesting that MAPKs pathway, especially ERK pathway, is truly activated in gentamicin‐induced HC apoptosis.

As STK33 suppression has been reported to induce cell apoptosis through MAPK pathway, we subsequently explored the effects of STK33 on MAPK pathway in HCs after gentamicin treatment. In the present study, we found that knockdown of STK33 only significantly inhibited STK33 expression but failed to change the p‐ERK1/2 expression in HEI‐OC1 cells after gentamicin treatment, whereas U0126 treatment, a MEK1/2 inhibitor, not only inhibited p‐ERK1/2 expression but also suppressed STK33 expression. The same phenomenon was also found in primary culture of cochlear HCs after gentamicin exposure. These results indicate that STK33 might be regulated by ERK1/2, and appear in the downstream of ERK1/2, which is consistent with our previous study in tumour cells.^13^

As ROS accumulation is generally related to aminoglycoside‐induced HC apoptosis^33^, ^34^ and MAPKs can be activated by ROS,^35^ we thus assessed the ROS levels in HCs. In this work, we found that ROS level was dramatically increased in cochlear HCs after gentamicin exposure, which is consistent with other researches.^36^ We also found that after gentamicin treatment, the ROS level was significantly increased in HEI‐OC1 cells with STK33 knockdown, which indicates that STK33 knockdown might increase the cellular ROS level to induce HC apoptosis. N‐acetyl‐L‐cysteine, which is an important precursor to many antioxidants, plays a key role in attenuating oxidative stress. In the current study, treatment with the ROS scavenger NAC rescued the increased mitochondrial dysfunction and apoptosis in HEI‐OC1 cells caused by STK33 down‐regulation after gentamicin injury. Together, STK33 knockdown leads to elevated ROS levels, which further contribute to apoptosis in HEI‐OC1 cells after gentamicin injury.

In conclusion, the findings from this work indicate that STK33 decreases the sensitivity to the apoptosis dependent on mitochondrial apoptotic pathway by regulating ROS generation after gentamicin treatment, which provides a new potential target for protection from the aminoglycoside‐induced ototoxicity. The exact mechanism(s) by which STK33 regulates the gentamicin‐induced impairment of inner ear of mice needs to be investigated further.

## CONFLICT OF INTEREST

The authors declare that there is no conflict of interest.
